# Object-Based Change Detection Algorithm with a Spatial AI Stereo Camera

**DOI:** 10.3390/s22176342

**Published:** 2022-08-23

**Authors:** Levente Göncz, András László Majdik

**Affiliations:** Machine Perception Research Laboratory (MPLab), Institute for Computer Science and Control (SZTAKI), Eötvös Loránd Research Network (ELKH), Kende u. 13-17, 1111 Budapest, Hungary

**Keywords:** robot perception, 3D mapping, object detection, change detection, mobile robot

## Abstract

This paper presents a real-time object-based 3D change detection method that is built around the concept of semantic object maps. The algorithm is able to maintain an object-oriented metric-semantic map of the environment and can detect object-level changes between consecutive patrol routes. The proposed 3D change detection method exploits the capabilities of the novel ZED 2 stereo camera, which integrates stereo vision and artificial intelligence (AI) to enable the development of spatial AI applications. To design the change detection algorithm and set its parameters, an extensive evaluation of the ZED 2 camera was carried out with respect to depth accuracy and consistency, visual tracking and relocalization accuracy and object detection performance. The outcomes of these findings are reported in the paper. Moreover, the utility of the proposed object-based 3D change detection is shown in real-world indoor and outdoor experiments.

## 1. Introduction

In this paper, we focus on detecting change in the environment. What is considered a change is defined by the given problem and can depend on, for example, the type of data and technology used or the time interval between inspections of the environment in which we are looking for changes. Change detection is also influenced by the purpose of analysis: patrolling robots used for security and satellites used by geologists interpret change differently.

Automated patrolling is becoming more and more prevalent by the increasingly available wheeled and legged mobile robots. These navigate with great mobility and their relatively cheap, intelligent cameras allow users to create more complex autonomous inspection tasks. Stereo cameras that come with various integrated sensors broaden the range of applications and make spatial analytics implementations easier and faster. In addition, the rapid evolution of neural networks in recent years has mad real-time inference possible.

Our goal was to create a real-time object-based 3D change detection method that could maintain an object-oriented metric-semantic map of the environment and detect changes between patrol routes captured at different times. The novel ZED 2 stereo camera [1] is an ideal platform for the proposed algorithm. This camera was built for the purpose of promoting and advocating the concept of spatial artificial intelligence (AI), which is the concept of augmenting spatial reasoning with deep learning, i.e., combining computer vision with AI [2]. It is the first stereo camera to combine 3D sensing with AI using built-in neural networks to help and expedite the development of computer vision perception systems.

The current state of the art in 3D change detection either compares multitemporal 3D point clouds directly [3,4], (3D-to-3D change detection), assumes that a 3D model of the scene is given and then use 2D images for change detection [5,6,7,8], (2D-to-3D change detection) or uses remote sensed, top-view data to detect environmental changes and changes on the Earth’s surface [9].

Our approach was inspired by methods using semantic object databases to reason about the environment on the level of objects. However, these methods are either more suitable for long-term change detection [10] and/or require a robust and complex system with a previous deep knowledge about the environment [11]. Our work goes beyond previous works by combining a fast and efficient method for building semantic object maps, (proposed by [12]) with the novel ZED 2 stereo camera for real-time change detection. The proposed solution is suitable for patrolling tasks thanks to the optimized and integrated combination of object detection, depth sensing and simultaneous localization and mapping (SLAM) features of the camera and the swift data association method of [12]. This combination enables real-time change detection and coherent 3D semantic map building.

To be able to design the change detection algorithm, the performance of the ZED 2 stereo camera is measured first. The results of the extensive set of tests with respect to depth measurement accuracy and consistency, object detection accuracy, SLAM accuracy and relocalization accuracy are reported in this paper. These metrics can be useful for anyone looking to do research and develop using the ZED 2 stereo camera.

To summarize, this paper advances the state of the art with the following contributions:

- We propose a state-of-the-art algorithm to detect object-level changes in real time between multitemporal patrol routes using the concept of metric-semantic object maps and the ZED 2 stereo camera.
- To design a suitable object detection system, the accuracy of the features of the ZED 2 stereo camera must be measured. We present the type of tests carried out and their results.
- We show the utility of the proposed object-based 3D change detection algorithm in real-world indoor and outdoor experiments, and we have released the source code for the algorithm (see in Supplementary Materials).

## 2. Related Work

Change detection is “the process of identifying differences in the state of an object or phenomenon by observing it at different times” [13]. There are many ways to categorize change detection methods, e.g., based on input data (e.g., satellite imagery, aerial photos, street-view photos, feature maps and three-dimensional point clouds [13,14]), technology and sensors used to acquire the data, methods used to analyze the data, etc.

Three-dimensional change detection is a subset of traditional remote sensing (RS) [15], which analyzes and determines the (typically long-term) changes of the surface from multitemporal images. However, in general, 3D change detection can be applied to both remotely sensed data captured from a top view or to close-range data that were captured near ground-level. Three-dimensional change detection is a quite recent field of study that was motivated by the increasingly available 3D information and also the need for it in, e.g., smart cities, more available autonomous vehicles and robots. The key difference between 3D change detection and traditional remote sensing is the additional dimension of input data. The data can be point clouds, 3D (city) models, digital surface models or stereo-view images that can express positions/shapes of objects along all three dimensions [16].

With the available 3D geometric information, the input data are not affected by illumination or perspective distortion, thus 3D change detection can provide a more accurate description of the environment. The commercialization and advancement of LiDAR systems, UAVs and stereo cameras and precise three-dimensional coregistration algorithms allow a cost-effective data acquisition and data processing. However, the quality and accuracy of 3D data depend highly on the precision of acquiring sensors and on the algorithm that is used during data gathering. Additionally, the change from 2D to 3D space leads to an increase in problem modalities, e.g., occlusions, incomplete data, point cloud registrations and 3D feature extractions [16].

### 2.1. State-of-the-Art 3D Change Detection Methods

Change detection methods usually have three main components: 1. data acquisition, 2. data coregistration/data association and 3. change analysis [16]. There are many forms of change detection methods, whose solutions differ in one or in all of these components [17]. Some approaches compare multitemporal 3D point clouds directly (3D-to-3D change detection). Girardeau-Montaut et al. [3] used a ground-based laser to scan the 3D environment at different times and defined a specific octree structure (an octree recursively subdivides the 3D space into eight smaller spaces), which was then used to compare various cloud-to-cloud comparison techniques. The result of the change detection was displayed as a heatmap. This method was highly sensitive to point-sampling variations between scans and the running time depended greatly on the octree level.

Fanfani and Colombo [4] also used a cloud-to-cloud comparison technique. Point clouds were generated from multitemporal image collections using the structure-from-motion (SfM) method. After generating multitemporal point clouds, the paper proposed to use a random sample consensus (RANSAC) framework for the estimation of the rigid transformation between point clouds using a previously generated 3D feature-match matrix. As a final step before change detection, an iterative closest point (ICP) algorithm was used to align the point clouds. The change detection algorithm worked by shifting a 3D box over the aligned point clouds and computing a majority voting scheme that compared the density, shape and distribution of 3D points. The result of the change detection was displayed as a heatmap. The change detection method proposed in that paper and most methods that are based on cloud-to-cloud comparison techniques are mainly used in postprocessing and are inefficient as real-time solutions with regard to time and computation.

There are several 3D change detection approaches that assume that a 3D model of the scene is given and then use 2D images for change detection (2D-to-3D change detection). Golparvar-Fard et al. [5] assumed that a building information model (BIM, which has information about geometry and relationships of elements) was given and utilized a 2D image collection to reconstruct a construction scene using structure-from-motion, multiview stereo (MVS, which is an extension of standard stereo vision to multiple images) and voxel coloring algorithms with a probabilistic framework. However, that method relied greatly on the quality of the obtainable MVS reconstruction and also on the availability and novelty of the 3D building information models.

Taneja et al. [6] and Taneja et al. [7] proposed a method to detect changes in the geometry of a city using cadastral 3D models provided by city administration and Google Street View panoramic images. The key idea was to obtain a pixel-wise map of inconsistencies between the geometry and two multitemporal images of the environment by projecting one of the images onto the other using the provided 3D geometry models. Then, based on this inconsistency map, the change detection algorithm estimated a binary labeling for each uniformly sized voxel in the entire grid of the 3D model. The results showed an increase in accuracy compared to similar, state-of-the-art solutions. However, due to reflections and occlusions, the number of false positives was relatively high. Moreover, the computation time was around 1 min per region, which made these solutions unsuitable for real-time change detection.

Instead of picking pairs of images, Palazzolo and Stachniss [8] proposed to combine multiple images, such that the 3D location of the change could be estimated with fewer ambiguities. Apart from being more accurate, the above-mentioned solution was suitable for real-time change detection as, according to the authors, the total computational time was less than two seconds. However, that solution built upon an already-built, dense 3D map of the environment. This meant that, during change detection, the 3D map was not being continuously updated; therefore, it was not perfectly suitable for patrolling tasks that require consecutive comparisons and continuously updated 3D models.

To reduce the computational time and to increase the accuracy of MVS-based change detection methods, Sakurada et al. [18] proposed a probabilistic framework to quickly detect damages of a city after earthquakes. The key idea in the paper was that the exact structure of a scene was not needed in order to detect changes. The estimation of the scene structure was done by using a probabilistic density of depths. Then, this density was used in such a way that the depth ambiguity was well reflected in the final change estimation. Their proposed method reduced computational time compared to other MVS-based methods, but still did not perform change detection in real time and therefore was inefficient for our purpose.

Andreasson et al. [19] proposed a change detection algorithm that used a 3D range sensor and a camera to estimate depth information only at the position of local visual features in the image. After registering the obtained multitemporal point clouds, change was detected using a probabilistic framework based on color and spatial difference. Their method was suitable for autonomous change detection with a security patrol robot; however, change analysis did not happen in real time and therefore it was not perfectly applicable to our algorithm.

Lawson et al. [20] proposed an algorithm that was between general and object-level change detection. The paper presented a method where the robot was fixed on a patrol route and tried to build a dictionary of the clustered features present in the scene. Later, based on this feature dictionary, the robot was able to detect scene-specific anomalous objects. The algorithm did not know any information about the objects, only detecting if a feature cluster had changed or not. Additionally, the change detection algorithm produced a very high number of false positives due to occlusions. The authors suggested a higher-level reasoning be added to their solution to identify the likely cause of change and to rule out false positives.

Shi et al. [9] surveyed the state-of-the-art change detection methods that are based on artificial intelligence. However, most of them use remotely sensed, top-view data to detect environmental changes and changes on the Earth’s surface and not ground-level information for real-time change detection.

### 2.2. 3D Semantic Object Maps

To design the most suitable 3D change detection algorithm for the ZED 2 stereo camera, research has also been focused on object detection based on semantic mapping.

There are novel publications that investigate the possibility of using object-oriented semantic maps to give higher-level data to robots. Semantic maps are able to describe the environment from an object-level perspective and give a higher-level reasoning to autonomous systems. Nüchter and Hertzberg [21] defined a semantic map as “a map that contains, in addition to spatial information about the environment, assignments of mapped features to entities of known classes. Further knowledge about these entities, independent of the map contents, is available for reasoning in some knowledge base with an associated reasoning engine”.

McCormac et al. [22] and Nakajima and Saito [23] proposed semantic mapping systems combining CNN-based semantic labeling and SLAM. The architecture used a real-time SLAM system for finding correspondences between frames and thus achieving localization. Separately, it also used a CNN network to return 2D pixel class probabilities for RGB or RGBD images. Finally, the output of these two parts were taken into consideration when updating the 3D map. The idea to combine two separate algorithms for semantic mapping is useful and can be incorporated into a change detection system. However, the publications did not discuss how to further use the semantic map for more manipulation and/or database design.

The system proposed by Sünderhauf et al. [12] gave a solution to the previously mentioned problem. It simultaneously built a 3D model of the environment and also created an object-oriented semantic map with a database. To provide more advanced scene understanding, bounding-box-based object detection and unsupervised 3D segmentation were combined with simultaneous localization and mapping. The building of the semantic map happened in a step called data association. It determined whether a detected object was already built into the map (hence only needing to update a previously saved map object) or whether it needed to be added as a new map object. The data association happened in two stages. For every detected object, the system queried the semantic object map to select previously saved objects whose point cloud centroids were below a predefined Euclidean distance threshold. Secondly, a nearest neighbor search was performed between the segmented 3D object point clouds (the detected object’s and the queried, already saved object’s point clouds) to calculate the Euclidean distance between the neighboring point pairs. If at least half of the associated 3D point pairs of the two object point clouds had a distance smaller than a predefined threshold, the detection was associated with an already-saved object in the semantic object map. According to their measurements, this process took around 30 ms for each comparison. Their work stopped at the built 3D semantic map and only gave future possibilities on how this map could be used. Still, this approach did not require previously built 3D models of the environment and the mapping and the building of the object database happened in real time.

Zhang et al. [24] developed an object detection based on a semantic SLAM system by using the YOLO object detector for real-time object detection and a SLAM system for simultaneous localization. The architecture proposed in that paper was similar to the one in [12], in that they both simultaneously traversed a 3D scene and built a semantic object database using the gathered data in real time. The main difference between the proposed system and the previously mentioned semantic mapping solutions is that it uses an octomap-based structure for storing the point clouds at every key frame. Using an octomap accelerates the speed of mapping, because regular point clouds make querying and searching a map inefficient. Another drawback of point clouds is that they cannot categorize, e.g., the empty areas, and cannot eliminate noise, which makes path planning and collision detection harder.

Truong et al. [25] also used an object detection algorithm on top of SLAM to obtain the semantic information of landmarks in the map. The proposed solution was suitable for dynamic environments and for building a semantic database, it used a triplet ontological semantic model (TOSM) database that was introduced by Joo et al. [11]. The goal in defining the TOSM database was to enable systems to carry out complex tasks using high-level and human-like knowledge. TOSM aims to mimic the brain’s GPS model efficiency by storing the metric information, geometrical features and image information of objects, but also relations between environmental elements.

Kunze et al. [10] proposed a semantic object-mapping framework called SOMa, designed to “map locations of objects, regions of interest, and movements of people over time”. They specifically introduced this framework to help researchers understand change in dynamic environments. Their open-source system is adaptive for changes, can be queried and stores objects and regions of interests over time. However, SOMa stores discrete observations of objects and is more suitable for long-term case studies, not real-time change detection.

In contrast to the aforementioned methods, we aimed to create a semantic map-based architecture that is suitable for real-time change detection: the semantic map should not require complex previous knowledge and heavy computations to maintain. Querying and updating the semantic map should happen efficiently in real-time and our method should be easily adaptable to any environment or use-case.

## 3. Assessment of the ZED 2 Stereo Camera

In this section, we present the tests that we carried out on the ZED 2 stereo camera and the results of these assessments. The datasheet of the camera reports limited information about the accuracy of the various features of the camera. Before designing a change detection algorithm using the camera, it is necessary to measure the quality and reliability of these characteristics of the camera.

To meet the recommended requirement specifications [26] and to be able to use all the features of the camera, a computer with an Intel Core i7 processor @2.7 GHz, 32 GB RAM and NVIDIA GeForce GTX 980 GPU with CUDA driver v11.1 was used during the development and experiments.

The following sections present the performance of the camera with respect to depth accuracy and consistency, visual tracking and relocalization accuracy and object detection.

### 3.1. Depth Measurement Accuracy

**Figure 1:**
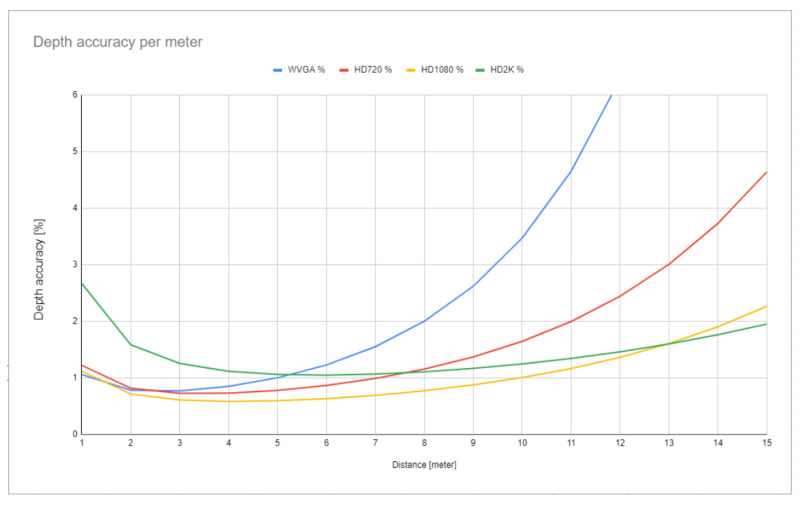
Depth accuracy of the ZED 2 stereo camera in percentages estimated by an exponential model described in [27,28] between 1 and 15 m.

The ZED 2 stereo camera has a reported depth range of 20 m and a depth accuracy of <1% up to 3 m and <5% up to 15 m [26]. To determine the usefulness and usability of the camera for real-time applications, it was important to measure more accurately its depth precision and depth measurement consistency. A 3D checkerboard-based test method described by Fernandez et al. [27] and Ortiz et al. [28] was used to assess the depth measurement accuracy of the camera. The accuracy was determined for all four available resolutions of the camera at every meter between 1 and 20 m. The results (displayed up to 15 m) are shown on Figure 1. At a distance of 5 m, the accuracy was measured to be below 1% for every resolution. The HD1080 resolution had a measured accuracy below 1% up to 10 m and below 2.26% up to 15 m. Note, at a distance of 15 m, the HD720 resolution had an accuracy of 4.64%, while it was 1.95% for the HD2K resolution. The WVGA resolution passed the 5% accuracy threshold already around 11 m and had an accuracy of 16% at a distance of 15 m, making it very unreliable at long distances.

### 3.2. Depth Measurement Consistency

The test method proposed by Ortiz et al. [28] was used to assess the depth measurement consistency of the camera. Two scenes were captured with a maximum depth of 6 m: one recorded indoors with artificial light and one outdoors with natural light. For all four resolutions of the camera, the standard deviation (std) of every pixel in the scene for the 100 consecutive depth maps was computed. For both scenes, the HD1080 resolution proved to be the most consistent one. As seen in Table 1, it had the lowest mean std, lowest median std and lowest maximum std values out of all four resolutions. In the outdoor scene, the WVGA and HD720 resolutions had a much higher maximum std value compared to the other two resolutions and the WVGA also had a mean std value of around 10 times higher compared to that of the HD1080 resolution, making it less reliable for depth sensing.

To have an estimate about the time needed for the ZED 2 stereo camera to acquire the depth map and 3D map of a scene, 50 consecutive tests were carried out in the same environment. For the results, please refer to Table 2.

To conclude, the outcome of these tests showed that with respect to depth measurement accuracy and consistency, the most reliable resolution and ideal design parameter of the 3D change detection algorithm is the HD1080 resolution. It requires 2.11 ms on average to obtain the depth map of a scene at its maximum (30) frames per second setting.

### 3.3. Visual SLAM Accuracy

**Figure 2:**
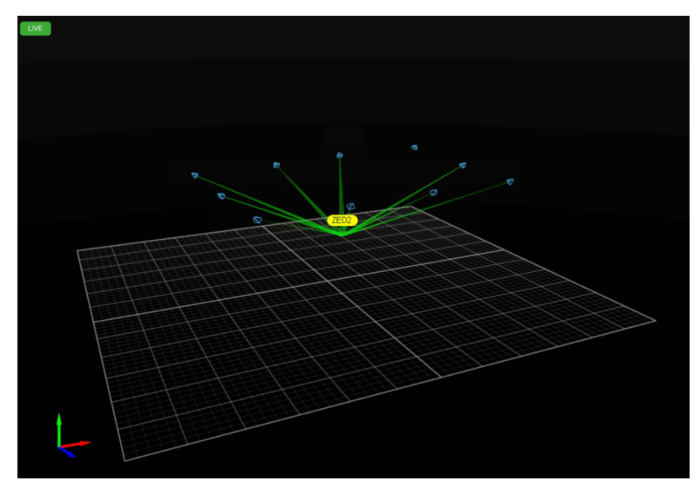
Tracking the movements of the ZED 2 stereo camera with the MIMO system.

The visual SLAM (simultaneous localization and mapping) system allows the camera to provide an accurate 3D representation of its environment while being able to continuously locate itself in it. To test the visual SLAM accuracy of the ZED 2 stereo camera, the SZTAKI MIMO (micro aerial vehicle and motion capture) Arena proposed by Rozenberszki and Majdik [29] was used. The tests carried out inside the Arena had a mean length of 12 m. The system was continuously tracking three retroreflective markers on top of the camera, as shown in Figure 2, and recorded the ground-truth trajectory and orientation, which were compared to the trajectory and orientation recorded by the ZED 2 stereo camera. To estimate the translation error, the root-mean-square (RMS) error was calculated between the ground truth and measured trajectories. Furthermore, the evaluation metrics of the KITTI Vision Benchmark Suite (KITTI) [30] were used.

The visual SLAM system of the ZED 2 stereo camera was measured to have an average of 0.184 deg/m rotation error (KITTI) and 3.073% translation error (KITTI). The average measured RMS translation error was 2%.

The positional tracking accuracy of the visual SLAM system of the ZED 2 stereo camera was also measured by comparing the difference between the 3D position reported by the camera at the beginning and at the end of a long test sequence. The test sequences were conducted indoors, using the HD1080 resolution of the camera. With an average trajectory length of 121.9 m, the average drift was measured to be 2.13 m. Averaging the test sequences, the ZED 2 stereo camera had an average drift of 1.75%.

### 3.4. Relocalization Accuracy

It was also important to measure the special relocalization ability of the ZED 2 stereo camera that enables it to share a fixed-reference world coordinate frame between different recordings of the same environment. The camera utilizes a so-called area memory for this task, which mimics human memory and incrementally stores the spatial information of the environment in a map [1]. This feature is very effective for fast change detection applications, because it enables the camera to locate itself in an already-visited area and detect changes more quickly and easily. The localization accuracy was also measured using the SZTAKI MIMO Arena [29]. The indoor tests were conducted with six different benchmark locations. The relocalization accuracy was measured as the discrepancy between the reported 3D positions of the ZED 2 stereo camera at these six benchmark locations of two consecutive recordings. The test sequences, after relocalization, had an average discrepancy of 66.71 mm.

### 3.5. Object Detection Accuracy

The ZED 2 stereo camera is capable of 2D and 3D object detection. The camera is able to use its proprietary object detector or can be interfaced with many well-known frameworks to detect objects of interest. However, the object detector of the ZED 2 stereo camera has no reported performance value associated with it. It is also proprietary, which means that its architecture is not accessible to users. Furthermore, it can only process input images that are coming from either a live video stream of the camera or from its specific .svo recording format. This makes using large online test image databases impossible for performance measurement. Nonetheless, in order to assess the performance of the ZED object detector, a qualitative test process was designed. Using the ZED 2 stereo camera, 51 images were captured (in HD1080 resolution) and the output of the object detector for each image was saved for further evaluation.

**Figure 3:**
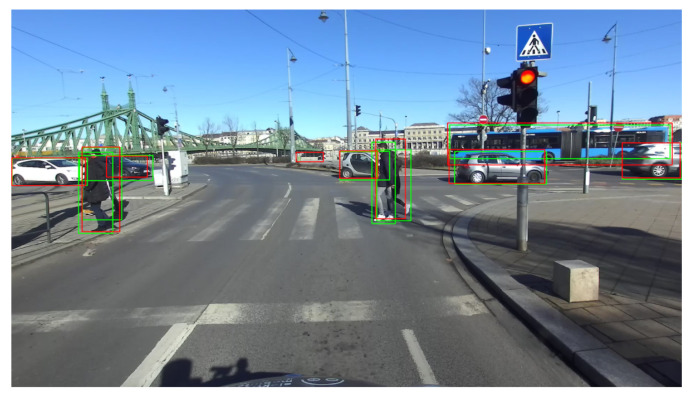
Example image of the output of the ZED 2 object detector generated using the toolkit proposed by Padilla et al. [31]. Bounding boxes of the detections are shown in red. Bounding boxes of the ground truth annotations are shown in green.

The image acquisition happened on the road while the camera was mounted onto a test vehicle (see Figure 3 for a real example), in a garden with various objects present and inside a house. The goal of the image acquisition was to gather images in a broad spectrum, representing as many object classes the ZED 2 object detector could recognize as possible. After the recording of a dataset of 51 images, an online annotation tool was used to label the classes on the captured images, thus creating the ground-truth data for the object detection performance assessment. In total, 342 objects were labeled in the 51 images, belonging to 16 different classes. The assessment of the object detection performance was done by using the toolkit proposed by Padilla et al. [31]. The qualitative test process concluded that the ZED object detector had a measured mean average precision (mAP) value of 0.53 (IoU = 0.5) and a mean inference time of 51.74 ms.

## 4. 3D Change Detection Algorithm

In our proposed algorithm, we utilized the semantic mapping framework proposed by Sünderhauf et al. [12] and used it for change detection with the ZED 2 stereo camera.

In general, change detection is the process of identifying changes between multitemporal observations. This means that, in effect, change detection compares two states inspected at different times. Along this idea, change detection algorithms have two main parts: at time t_i_, the environment has to be discovered and somehow registered in order to use the data gained through the exploration for change detection by comparing it with the discovery at time t_i+1_. Consequently, the change detection algorithm described in this paper had two main parts: 1. initial environment exploration and 2. change detection.

### 4.1. Initial Environment Exploration

**Figure 4:**
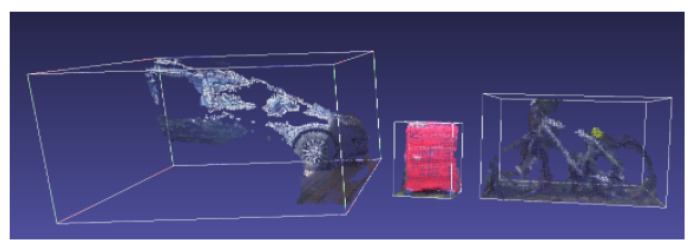
Example of 3D segmented point clouds of three objects: a car, suitcase and bicycle.

Algorithm 1 describes the strategy used for initial environment exploration. During a patrol route at time t_i_, the change detection algorithm, using the ZED 2 stereo camera, continuously builds a 3D spatial map, a semantic object map and a ZED-specific area map, which contains key features of the environment. Using this area map at the beginning of a consecutive patrol route at time t_i+1_, it is possible to maintain a shared-reference world coordinate frame across patrol routes. As the camera moves and explores its environment, the ZED object detector is continuously scanning for available objects in the scene. Once an object is found, the following attributes are extracted:

- Object label—one of the 22 object classes the ZED can recognize;
- Object ID—the camera tracks objects as long as they are in its field of view (FOV) and assigns them the same object ID;
- Detection Confidence—shows how confident the object detector is about the label of the detected object;
- Two-dimensional bounding box—2D pixel coordinates of the four corners of the two-dimensional rectangle that encapsulates the object in the 2D left image of the stereo camera;
- Three-dimensional bounding box—combining the four corners of the 2D bounding box with the depth map of the scene results in the eight 3D coordinates of the three-dimensional cube that encapsulates the object;
- Three-dimensional position—the 3D coordinates of the object’s centroid;
- Segmented 3D point cloud—using the 3D bounding box of a detected object, it is possible to segment the 3D point cloud that belongs to the object from the 3D point cloud of the scene (see Figure 4 for a real example);

**Algorithm 1** Initial environment exploration.1:**while** patrol_route **do**2:    Continuous 3D mapping and area map building of the environment3:    **for** all detected objects in the scene object_id=1,2,…,N **do**4:        Extract object features5:        Perform data association6:        Update metric-semantic database7:    **end for**8:**end while**9:Save metric-semantic database of the environment

**Figure 5:**
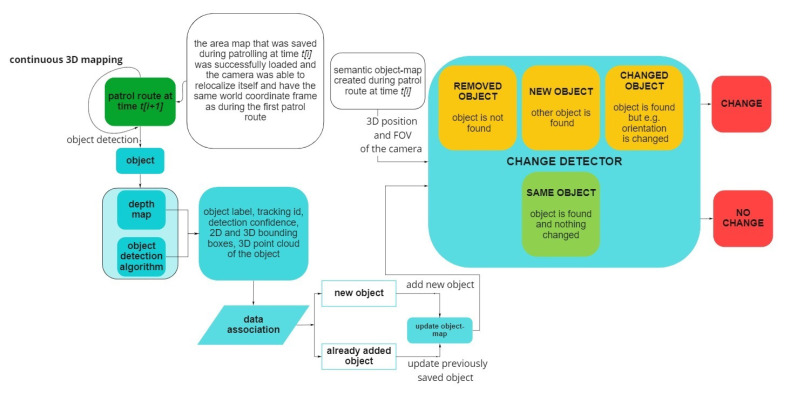
The flowchart of 3D semantic object map building and the object-map-based change detection algorithm.

Following the object detection, the data association step (based on the method proposed by Sünderhauf et al. [12]) determines if the detected object is an already-seen and registered object or a new, yet undetected and unsaved object. To decide this, the semantic object map the algorithm is building is queried to return those already-registered objects that are closest to the detected object. This step is done by querying the 3D positions of the objects’ centroids and calculating the Euclidean distance between the closest, already-registered objects and the newly detected object. Those already-registered objects, whose centroids are closer to the detected object than a predefined threshold, are obtained. If there are no close objects already stored in the object map, the detected object is added as a new object. Otherwise, a nearest neighbor search based on a k-d tree structure is performed between the detected object’s segmented 3D point cloud and a queried, already-registered object’s point cloud to calculate the Euclidean distance between the neighboring point pairs. If at least 50% of the associated 3D point pairs of the two point clouds have a distance smaller than a predefined threshold, the detected object is associated with the already-registered object in the semantic object map. Otherwise, the detected object is registered as a new object to the object map (as shown in the flowchart in Figure 5). The data association step takes on average 15 ms per detected object. With every stored object in the semantic database, a label–confidence and a label–number of detections pair is associated. This helps determine the final label and confidence score of every object in the semantic database.

At the end of a patrol route at time t_i_, a postprocessing step determines the final state of the semantic object map. A stored object’s label is decided by the label–confidence pair. The label with the highest accumulated confidence score is kept and based on the label–number of detections pair, the average confidence score for the object is calculated.

**Figure 6:**
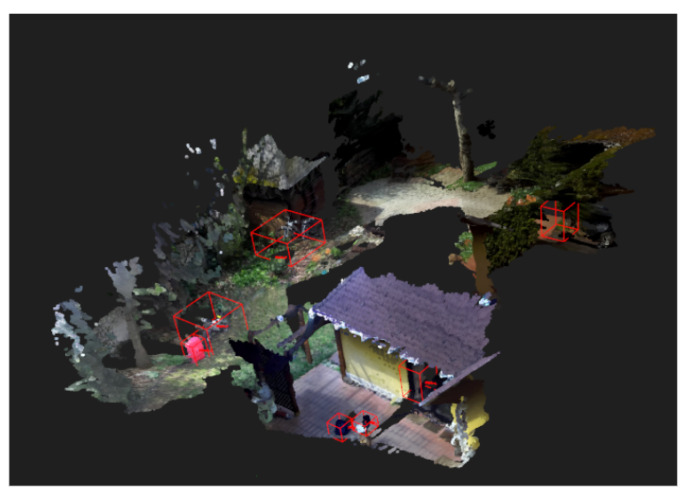
Result of the semantic object map after postprocessing with the 3D bounding boxes of objects displayed.

After postprocessing, the final semantic object map is stored in an XML tree structure, containing the path to the final 3D point cloud of the entire patrol route and to the area map constructed during mapping. Furthermore, it also stores the following attributes for every object in the database: object ID, object label, object detection confidence, number of detections of the object, 3D coordinates of the object’s centroid in the world coordinate frame, path to the 3D point cloud of the object, 2D bounding box coordinates of the object and 3D bounding box coordinates of the object in the world coordinate frame. For a real-world example of the final 3D point cloud of a complete patrol route combined with 3D bounding boxes of detected objects, see Figure 6.

### 4.2. Detecting Changes by Using the Semantic Object Database

Algorithm 2 describes the strategy used for detecting changes. At the beginning of the patrol route at time t_i+1_, the previously saved object map is loaded. This allows the algorithm to use the previously constructed area map to create a shared-reference world coordinate frame between the patrol routes. The objects in the semantic object database are also loaded and are used to look for changes in the environment.

The spatial mapping of the ZED 2 stereo camera during patrol route at time t_i+1_ starts after the camera successfully relocated itself using the area map. Once the world coordinate frames of the two consecutive patrol routes are aligned, the steps described in the initial environment exploration section are continuously repeated, including the data association step. This is needed to maintain an up-to-date semantic map of the environment and recognize changes later compared to the current patrol route.

While building the new semantic object map, at each new camera frame, the semantic object map recorded during the previous patrol route is being queried repeatedly based on the current 3D FOV (field of view) of the stereo camera.
**Algorithm 2** Change detection using the metric-semantic database of the environment.  1:Load previous metric-semantic database of the environment  2:Create shared-reference world coordinate frame between patrol routes  3:**while** patrol_route **do**  4:    Continuous 3D mapping and area map building of the environment  5:    **for** all detected objects in the scene object_id=1,2,…,N **do**  6:        Extract object features  7:        Perform data association  8:        Update current metric-semantic database  9:        Compare previous and current metric-semantic databases10:      Change detection11:    **end for**12:**end while**13:Save current metric-semantic database of the environment

**Figure 7:**
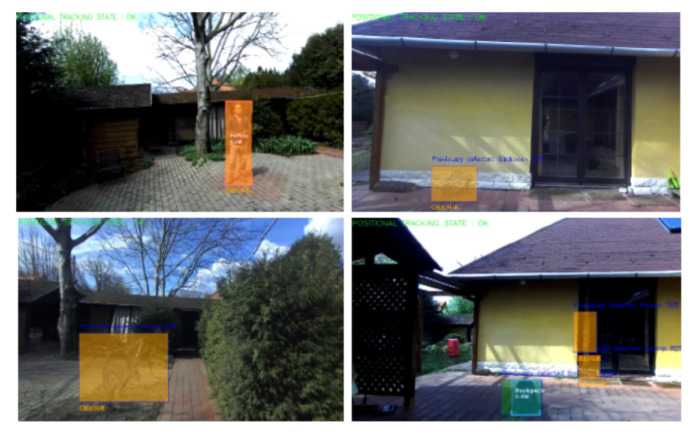
Examples of changes due to removed or added objects (shown in orange overlay). For changes caused by removed objects, the point clouds of previous detections are overlaid on the 2D image (top right, bottom left and bottom right example).

For every previously detected object that is in the 3D FOV of the camera, the algorithm returns the closest objects in the current semantic object map, using the Euclidean distance of 3D object centroid coordinates. If there are no objects in the current semantic object map that are closer than the predefined threshold, that means the object has been removed between the two patrol routes. Therefore, a change has occurred. Otherwise, the same nearest neighbor search is conducted to compare the point clouds of candidate objects as during the initial environment exploration. If at least 50% of the associated 3D point pairs of the two point clouds have a distance smaller than a predefined threshold, the objects are considered a match. If the 50% mark is not reached, it means that the point cloud of the two compared objects differ too much, that is, a change has occurred, because potentially another object has been placed at the same location or the object has changed between two patrol routes. Similarly, to detect new objects that appear during the current patrol route, but not the previous, the objects of the current semantic object map are queried. If they have no close objects in the previous semantic object map, a change has occurred. For real-world examples of change detection, see Figure 7.

## 5. Qualitative Performance Assessment of the Change Detection Algorithm

Due to the lack of existing .svo file-format-based databases, the performance of the change detection algorithm was measured with a qualitative test assessment. The two threshold values of the data association algorithm were empirically adjusted and during testing the Euclidean threshold was set to 1 m, while the threshold of the nearest neighbor search was set to 10 cm. Note that the Euclidean threshold value was not reported, and the threshold of the nearest neighbor search was set to 2 cm in Sünderhauf et al. [12]. However, the threshold of 2 cm was found to be too low in an outdoor setting (the work of Sünderhauf et al. [12] was based on a controlled, indoor office environment), thus it was increased in our change detection algorithm. The tests were carried out both in outdoor and indoor environments with various objects and in diverse scenarios, such as with the same object, removed object, changed object and added object.

The change detection algorithm could successfully and accurately detect changes that happened because a previously detected object had disappeared, or a new object had appeared in a place where there was no object before. In these cases, the algorithm did not carry out a comparison, because there were no close objects nearby. The algorithm could also handle objects closer to each other than the one-meter Euclidean distance threshold. The algorithm was able to find all the changes and matches in the three tests that were carried out with an average of nine objects each. However, it had difficulties when the detected objects were partially occluded or were too close to the camera (below 1 m). In these cases, the accuracy of the change detection algorithm dropped and because of the partially available point clouds of objects, it detected changes even though the objects did not move compared to the previous patrol route. In the future, one of the main goals of the change detection algorithm development will be to investigate the issue of occluded and close objects.

**Figure 8:**
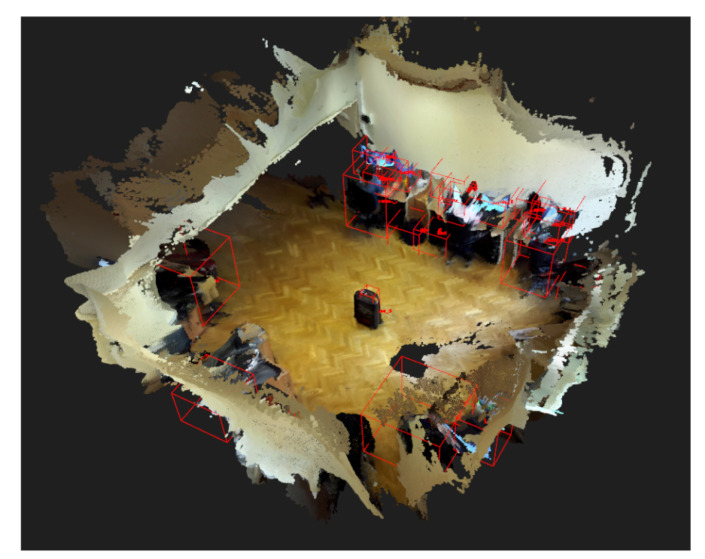
The 3D semantic object map of the SZTAKI office and the suitcase in the middle of it.

In order to show and assess the capabilities of the semantic-object-map-based change detection algorithm, a suitcase was placed in the middle of an office (as shown in Figure 8). The suitcase was recorded by walking around and capturing it from all possible angles. Following the initial environment discovery and semantic object map building, the same patrol route was carried out.

When the suitcase was at the same location with the same orientation, the algorithm successfully detected no change in the object between the two patrol routes. During the third patrol route in the same office area, the suitcase was moved to various locations. First, it was moved by around 5 cm, then 10 and 20. In the first two cases, the algorithm did not detect changes. This happened because the threshold of the nearest neighbor search was set to 10 cm beforehand. Therefore, translation changes that were below 10 cm were not registered as changes by the algorithm. However, during the last trial, when the translation was around 20 cm, the algorithm was able to detect the change in the location of the suitcase.

The change detection algorithm does not perform any 3D object point cloud alignment, e.g., an iterative closest point (ICP)-based algorithm, to check whether a point cloud of a rotated object belongs to a previously detected object. Therefore, it is not rotation-invariant and is sensitive to changes of orientation of objects. To measure this sensitivity, in this test case, three retroreflective markers were placed on the top of the suitcase. Using the SZTAKI MIMO Arena [29], the suitcase was rotated until the algorithm no longer recognized it as a matching object between patrol routes. The sensitivity of the change detection algorithm depended greatly on the object in question. Since the stored features in the object map are 3D point cloud points, the point cloud density and the object’s texture influenced how susceptible the algorithm was to a change in the orientation of objects. For the above-presented suitcase, the change detector did not detect changes even when it was rotated by 90°. The reason for this is that the texture of the suitcase was very simple and similar on all sides. Moreover, the 3D shape of the suitcase was also simple and when rotated, it did not change that much. On the other hand, with a more complex object, such as a bicycle, the change detector was more sensitive to changes and detected smaller rotations too.

## 6. Discussion

The tests carried out on the ZED 2 stereo camera gave us a wide range of data with respect to its performance. We hope that the results of these tests with respect to depth accuracy and consistency, visual tracking and relocalization accuracy and object detection performance can be used by anyone working with the camera. Some of the testing methods required special, accurate pieces equipment such as the SZTAKI MIMO Arena [29] and their results can be further used to compare this camera with other state-of-the art devices.

During our experiments with the ZED 2 stereo camera, we were relying on its specific file format (.svo) for storing videos along with additional metadata such as timestamp, depth and sensor data (temperature, magnetometer, barometer gyroscope and accelerometer). Therefore, for an evaluation, the test environment must be recorded with the ZED 2 camera, since the change detection algorithm relies on the various features stored in the recording. This specific input format poses a limitation on the change detection algorithm since it is only compatible with the ZED 2 stereo camera.

The experiments also showed that the speed of objects did not affect the change detection accuracy because the algorithm could effectively detect both missing and new objects (if the object changed position rapidly between two camera frames, a change was registered). The number of objects in the environment, however, directly influenced the time needed for the data association step (15 ms per object). Therefore, for the change detection process, a crowded environment with many objects in close proximity to each other is a limiting factor.

The qualitative experiments showed that the algorithm was capable of real-time change detection at the object-level. To compare our method with other state-of-the-art change detection methods, the accuracy of the algorithm will be measured in the future with a more representative, quantitative assessment.

## 7. Conclusions and Future Work

To conclude, this paper summarized the state-of-the-art 3D change detection methods. Furthermore, the paper also presented the novel ZED 2 stereo camera and the results of the extensive set of tests on the camera, which can provide a good benchmark for other researchers who are considering using the camera. The results of the tests showed that the ZED 2 stereo camera was an effective and reliable device for various real-time tasks requiring spatial AI capabilities.

Finally, the paper proposed a 3D change detection algorithm suitable for the ZED 2 stereo camera. The algorithm was inspired by the concept of 3D semantic object maps, especially by the work of Sünderhauf et al. [12]. The real-time 3D change detection algorithm was capable of detecting object-level changes in the environment and build a 3D object-oriented metric-semantic map at the same time. Our framework is easily adaptable to any environment and does not require and previous knowledge of the surrounding objects. The database requires low memory and can be swiftly loaded during change detection.

In the future, since the algorithm was only tested qualitatively, the performance of the change detection algorithm will also be more thoroughly measured with a quantitative assessment and an investigation will be conducted into the possibility of improving the current state of the algorithm, such as the handling of partially occluded and close objects. Furthermore, the data association step will also be updated to not only rely on the segmented 3D point cloud of objects, but their additional features, such as color and point cloud density. This will help provide a more detailed change detection result. The speed of the data association step will also be improved to make the change detection algorithm faster and therefore more robust. 

## Figures and Tables

**Table 1:** Standard deviations of every pixel in the scene for the 100 consecutive depth maps in all four resolutions for the indoor and outdoor scene.

Resolution	Mean (m)	Median (m)	Maximum (m)
WVGA	0.0436 and 0.3696	0.0207 and 0.0267	3.1053 and 9.8983
HD720	0.0409 and 0.0805	0.0296 and 0.0139	3.3151 and 9.1521
HD1080	0.0361 and 0.0336	0.0107 and 0.0082	3.0960 and 6.1823
HD2K	0.0370 and 0.0641	0.0111 and 0.0083	3.1041 and 6.6811

**Table 2:** The time needed to obtain the depth map and point cloud of a scene, calculated by averaging 50 consecutive depth and point cloud retrieval tests for 4 resolutions with their maximum possible frame rate.

Resolution and Max. FPS	Depth Map Time (ms)	3D Point Cloud Time (ms)
WVGA—100 FPS	0.356	0.829
HD720—60 FPS	0.960	2.934
HD1080—30 FPS	2.113	6.396
HD2K—15 FPS	2.860	8.841

## Data Availability

Not applicable.

## Supplementary Materials

Please note that this paper is accompanied by the source code of the proposed algorithm and its documentation, publicly available at: https://github.com/nyakasko/ZED_2_change_detection (accessed on 19 August 2022).
